# Cultivar Differences in Root Nitrogen Uptake Ability of Maize Hybrids

**DOI:** 10.3389/fpls.2017.01060

**Published:** 2017-06-20

**Authors:** Qiang Li, Yawei Wu, Wei Chen, Rong Jin, Fanlei Kong, Yongpei Ke, Haichun Shi, Jichao Yuan

**Affiliations:** Department of Crop Cultivation and Farming System, College of Agronomy, Sichuan Agricultural UniversityChengdu, China

**Keywords:** maize, root, nitrogen uptake, kinetic parameter, cultivar

## Abstract

Although, considerable differences in root size in response to nitrogen (N) application among crop species and cultivars have been widely reported, there has been limited focus on the differences in root N uptake ability. In this study, two maize (*Zea mays* L.) hybrids, Zhenghong 311 (ZH 311, N-efficient) and Xianyu 508 (XY 508, N-inefficient), were used to compare differences in root N uptake ability. The two cultivars were grown in field pots Experiment I (Exp. I) and hydroponic cultures Experiment II (Exp. II) supplemented with different concentrations of N fertilizer. In both experiments, the levels of accumulated N were higher in ZH 311 than in XY 508 under low- and high-N supply, and the increment in accumulated N was greater under N deficiency. The maximum N uptake rate (*V*_m_) and average N uptake rate (*V*_a_) in Exp. I, the root N kinetic parameter maximum uptake rate (*V*_max_) per fresh weight (FW) and *V*_max_ per plant in Exp. II, and the root N uptake rate in both experiments were significantly higher for ZH 311 than for XY 508. In contrast, the root-to-shoot N ratio in both experiments and the root N kinetic parameter Michaelis constant (*K*_m_) in in Exp. II were markedly higher in XY 508 than in ZH 311, particularly under N-deficient conditions. Higher root N kinetic parameters *V*_max_ per FW and *V*_max_ per plant and lower *K*_m_ values contributed to higher N affinity and uptake potential, more coordinated N distribution in the root and shoot, and higher root N uptake rates throughout the growth stages, thus enhancing the N accumulation and yield of the N-efficient maize cultivar. We conclude that the N uptake ability of roots in the N-efficient cultivar ZH 311 is significantly greater than that in the N-inefficient cultivar XY 508, and that this advantage is more pronounced under N-deficient conditions. The efficient N acquisition in ZH 311 is due to higher N uptake rate per root FW under optimal N conditions and the comprehensive effects of root size and N uptake rate per root FW under N deficiency.

## Introduction

Maize (*Zea mays* L.) is not only a key component in human and animal diets worldwide but is also an important energy crop and a raw material in the food industry (Schnable et al., 2009; Yin et al., 2014). Maize is grown on 177 million ha of land worldwide and its total yield exceeds that of all other grains (FAO, 2012). However, there are two major challenges facing corn producers: (1) improving grain yield to satisfy increasing human requirements, and (2) increasing nitrogen (N)-use efficiency for sustainable agriculture (Tilman et al., 2011; Mueller et al., 2012; Chen et al., 2014a). In China, maize yield has increased steadily from 3.12 t ha^−1^ in 1980 to 5.81 t ha^−1^ in 2014 (Vitousek et al., 2009; National Bureau of Statistics of China, 2015). However, over the last 10 years, maize production has either declined or stagnated in most provinces in China, despite a linear increase in fertilizer consumption (Jia et al., 2014). Consequently, China has become the largest consumer of N fertilizer in the world, accounting for 9% of the world's arable land and more than 33% of the world's consumption of N fertilizer (Liu et al., 2013; Chen et al., 2014a). In China, the average N-use efficiency (NUE) in maize decreased from 35.0 kg kg^−1^ in 1,980 to 11.4 kg kg^−1^ in 2014. In contrast, maize NUE increased from 39.4 to 53.2 kg kg^−1^ in the USA during the same period (Vitousek et al., 2009; National Bureau of Statistics of China, 2015). It has been well-documented that maize yield and NUE can be enhanced by agricultural practices that minimize N leaching (Abbasi et al., 2013; Zhao et al., 2013), such as deep placement of urea and application of slow-release or controlled-release N fertilizers, as well as by developing N-efficient maize cultivars (Worku et al., 2007; Mu et al., 2015).

Breeding and using N-efficient maize cultivars is the most feasible way to increase maize grain yield and enhance NUE. Genotype differences in NUE have been reported in rice (Chen et al., 2015), maize (Mu et al., 2015), wheat (Singh et al., 2015), oilseed (Koeslin-Findeklee et al., 2015), potato (Tiemens-Hulscher et al., 2014), and barley (Hill et al., 2016). Maize genotypes can differ in their NUE, which is defined here as the ability of a genotype to produce superior grain yields under low N conditions in comparison with other genotypes (Presterl et al., 2002; Wang et al., 2004). Li et al. (2010) reported that the dry matter weight, N absorption, and grain yield of N-efficient maize cultivars were significantly higher than those of N-inefficient maize cultivars at the same N level in soil. Presterl et al. (2002) found that adaptation of hybrids from European elite breeding material to conditions with reduced N input was possible and mainly the result of an increase in N-uptake efficiency. The value of NUE is defined by N-uptake efficiency and N-utilization efficiency (Wang et al., 2004). N-uptake efficiency refers to the quantity of N absorbed by the plant relative to the available N in soil. In maize, N-uptake efficiency is regarded as a more important factor than N-utilization efficiency under N deficiency (Moll et al., 1982; Han et al., 2015). Therefore, it is essential to focus on differences in the N uptake ability of roots in maize cultivars with contrasting NUE under low N conditions.

As an integral part of plants, roots are involved in the acquisition of nutrients and water; synthesis of plant hormones, organic acids, and amino acids and anchorage. Moreover, they are the main interface between a plant and its soil environment (Hochholdinger and Tuberosa, 2009; Tsukagoshi, 2016). An essential strategy for improving NUE is to enhance N uptake by crops through breeding for suitable root traits. Effective N acquisition is not only dependent on the size of roots but also on the N uptake ability per root unit (Chen et al., 2014b; Mori et al., 2016). Chun et al. (2005) suggested that N-efficient cultivars took up more N and had greater root dry weight both with and without N supply than did N-inefficient cultivars, and proved that root size was the dominant factor determining N accumulation. Wang et al. (2004) reported that a larger root system (total root length and root surface area) contributes to efficient N accumulation in N-efficient maize cultivars when compared with N-inefficient maize cultivars.

Many researchers have reported differences in the root morphological characteristics of N-efficient and N-inefficient maize cultivars (Wang et al., 2004; Liu et al., 2009). However, there are few systematic reports regarding the N uptake ability of roots, which is the key factor in determining the N absorption differences between N-efficient and N-inefficient maize cultivars, particularly in the subtropical region of Southwest China. In our previous study, we showed that Zhenghong 311 (ZH 311) is an N-efficient cultivar with high yield and N uptake, whereas Xianyu 508 (XY 508) is an N-inefficient cultivar with low yield and N uptake (Li et al., 2016a,b). In the present study, we conducted a 2-year field pot experiment and a hydroponic culture experiment, in which plants were supplied with different levels of N, using the maize cultivars ZH 311 and XY 508 to investigate the amount of N uptake, root N uptake rate, root-to-shoot N ratio, and N uptake kinetics. The specific objectives of this study were as follows: (1) to evaluate the effects of different levels of N supply on the amount of N uptake, root N uptake rate, root-to-shoot N ratio, and N uptake kinetics; (2) to investigate the differences in root N uptake ability between N-efficient and N-inefficient maize cultivars; and (3) to identify the key factors determining the N absorption differences between N-efficient and N-inefficient maize cultivars.

## Materials and methods

### Experimental conditions

**Figure 1:**
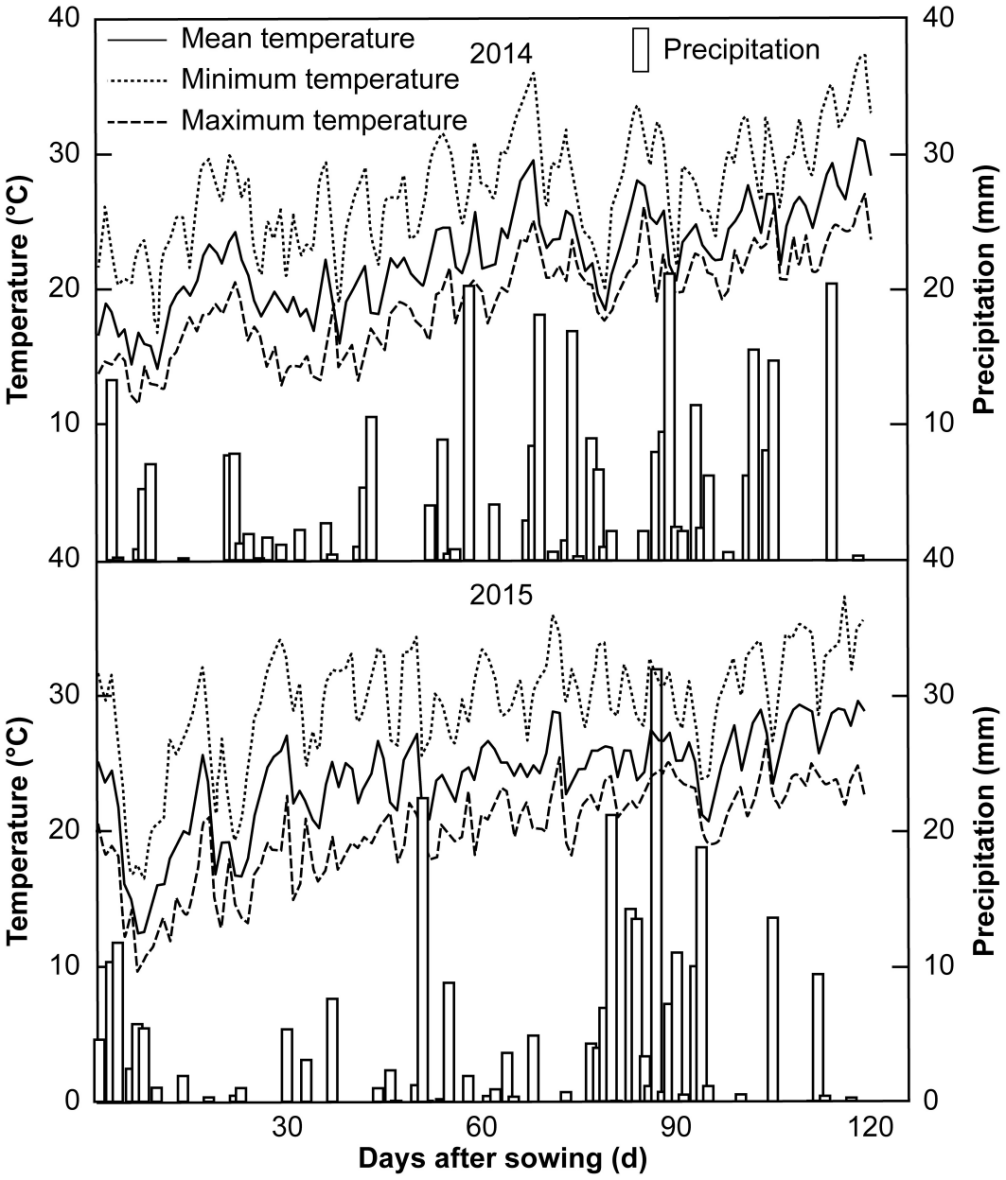
Climate data for Jianyang during the experimental period.

This study comprised two experiments conducted in two different regions over a span of 2 years. Experiment I (Exp. I) was a field pot experiment conducted in Jianyang, Sichuan Provence, China (30°38′N, 104°53′E and 429 m altitude), during the 2014 and 2015 growing seasons, whereas experiment II (Exp. II) was a hydroponic culture experiment carried out in Wenjiang, Sichuan Province, China (30°71′N, 103°87′E and 538 m altitude), during the 2015 growing season. The experimental soils for Exp. I were collected from the top 20-cm layer in a field with long-term maize production. The soil was air-dried and sieved through a 0.5-cm mesh, and visible roots and organic residues were removed manually. The soil was a typical purple soil with the following chemical compositions in 2014 and 2015 (the values for 2015 are listed parenthetically): 15.75 (13.30) g·kg^−1^ organic matter, 1.75 (1.56) g·kg^−1^ total N, 0.57 (0.40) g·kg^−1^ total P, 12.61 (8.25) g·kg^−1^ total K, 39.26 (36.34) mg·kg^−1^ alkali-hydrolyzable N, 2.55 (2.27) mg·kg^−1^ Olsen-P, and 139.33 (128.50) mg·kg^−1^ exchangeable K. The daily air temperature and precipitation recorded in Jianyang during Exp. I are shown in Figure 1.

### Experimental design

The design of Exp. I was a randomized block with three replicates. The two hybrids (ZH 311 and XY 508) and three N rates in 2014 (0, 150, and 300 kg ha^−1^) and four N rates in 2015 (0, 150, 300, and 450 kg ha^−1^) were randomly assigned to each replicate. The experiment was carried out in plastic pots with a mean diameter of 30 cm and height of 30 cm. Each replicate consisted of 20 pots, resulting in a total of 360 pots in 2014 and 480 pots in 2015. Each pot was filled with 20 kg of soil and arranged in alternating wide and narrow rows (1.4 m + 0.4 m) equivalent to a maize density of 52,500 ha^−1^. The seedlings were germinated in a nursery and those with at least two fully expanded leaves were transplanted at a density of two seedlings per pot. All pots were supplied with 72 kg·ha^−1^ of P_2_O_5_ as a single superphosphate and 90 kg·ha^−1^ of K_2_O in the form of potassium chloride as basal fertilizer. N fertilizer in the form of urea was equally split-applied as basal fertilizer and supplementary fertilizer. The management of all other aspects of plant cultivation was identical for each plot in both years. Water, weeds, insects, and disease were controlled as required to prevent grain yield loss.

The design of Exp. II was a complete randomized block. Maize seeds (ZH 311 and XY 508) were surface-sterilized in 10% (v/v) H_2_O_2_ for 40 min, washed five times with distilled water, and soaked for ~12 h in saturated CaSO_4_ solution with continuous aeration supplied by an electric pump. The seeds were germinated at 28°C under a 14/10 h light/dark photoperiod. When two leaves were fully expanded, the endosperm was removed and uniform seedlings were transferred into black plastic pots (20 seedlings per pot) containing 10 L of nutrient solution. The basic nutrient solution consisted of 0.75 mM K_2_SO_4_, 0.1 mM KCl, 0.25 mM KH_2_PO_4_, 0.65 mM MgSO_4_, 0.13 mM EDTA-Fe, 1.0 μM MnSO_4_, 1.0 μM ZnSO_4_, 0.1 μM CuSO_4_, and 0.005 μM (NH_4_)_6_Mo_7_O_24_. Seedlings were randomly divided into a normal-nitrogen group (supplied with 4 mM N; CK) and a low-nitrogen group (supplied with 0.04 mM N; LN). Ca(NO_3_)_2_ was used as a nitrogen source, and Ca^2+^ deficiency was rectified by supplementation with CaCl_2_ in LN treatments. The seedlings were grown in a growth chamber at 28/22°C under a 14/10 h light/dark cycle. The nutrient solution was renewed every third day and aerated continuously using an electric pump. The pH was adjusted to 6.0 with KOH.

### Sampling and measurements

In Exp. I, at the jointing (JS), large bell (LBS), silking (SS), filling (20 days after silking, FS), and maturity (MS) stages, samples were collected from two neighboring pots (four plants), and each sample was divided into root, leaf lamina, stem plus sheath, and panicle (grain and bract plus cob at the MS). Fresh samples were oven-dried at 105°C for 1 h and then at 80°C until constant weight to determine dry matter accumulation in different organs. The dried samples were ground and sifted through a 0.5-mm griddle to determine the total N concentration using an automatic Kjeldahl apparatus (Kjeltec-8400; Foss, Sweden).

In Exp. II, 18 days after N treatment, 10 uniform seedlings were sampled from each treatment (with three replicates), and each sample was divided into root and shoot. Dry matter accumulation and total N concentration were determined following the protocol described for Exp. I. Kinetic parameters were assessed 9 days after N treatment using the depletion method. Uniform seedlings were sampled and washed, initially with running water and then with deionized water. The plants were then soaked in 0.2 mM CaSO_4_ solution for 1 day to eliminate the effect of residual N in free space. (NH_4_)_2_SO_4_ and NaNO_3_ solutions were used at seven different N concentrations (0.05, 0.1, 0.2, 0.4, 0.6, 1.0, and 2 mM) and pH was adjusted to 6.0. Five uniform maize seedlings were selected as one replicate, and the experiments were conducted in three replicates for each N concentration. The seedlings were subsequently soaked in 250 mL of N solution for 2 h in an illuminated incubator at a light intensity of 4,000 lx and 25°C. After soaking, the roots were cut off, dried with filter paper, and weighed. The content of NH_4_-N and NO_3_-N in the roots was analyzed by using an automated continuous flow analyzer (FUTURA; AMS Alliance, Frépillon, France). The net N absorption rate of the root in unit time was calculated from the change in concentrations before and after absorption.

### Calculation methods

The logistic equation is a sigmoidal curve that can be used to model crop growth. It has been widely used to assess biomass yield, crop height, and leaf area expansion (Sheehy et al., 2006; Yan et al., 2006; Sepaskhah et al., 2011). Herein, it was used to fit the maize N uptake curve. Using the logistic equation, we can calculate the final theoretical N accumulation (*M*), maximum N uptake rate (*V*_m_), average N uptake rate (*V*_a_), time of instantaneous maximum slope (*T*_0_), time of N uptake rate acceleration (*T*_1_), time of N uptake rate deceleration (*T*_2_), and rapid N uptake period (*T*_2_–*T*_1_) as follows:

$$\begin{array}{l} \text{y}=\frac{M}{\left(1+aEXP^{-\text{bx}}\right)}\\ \text{Vm}=\frac{Mb}{4}\\ \text{Va}=\frac{M}{Growth\;period}\\ \text{T}0=\;\frac{\sqrt{a}}{b}\\ \text{T}1=\frac{lna-1.317}{b}\\ \text{T}2=\frac{lna+1.317}{b} \end{array}$$

where *x* represents the number of days after sowing, *y* represents N accumulation, and a and b are the constants of the fitted equation.

The root N uptake rate and root-to-shoot N ratio were calculated as follows (Liu et al., 2009):

$$\begin{array}{l} \text{Root N uptake rate}\\ =\frac{N\;accumulation\;at\;t2-N\;accumulation\;at\;t1}{Root\;dry\;weight\;at\;t2+Root\;dry\;weight\;at\;t1}\times 2\\ \text{Root to shoot N ratio}=\frac{\left(Root\;N\;accumulation\right)}{\left(Shoot\;N\;accumuation\right)} \end{array}$$

The N absorption kinetics curve was fitted according to the Michaelis–Menten equation (Xu et al., 2014; Xiaochuang et al., 2015):

(1) $$\begin{array}{l} \text{V}=\frac{Vmax\;C}{Km+C} \end{array}$$

where *V* is the uptake rate, *V*_max_ is the maximum uptake rate, *K*_m_ is the apparent Michaelis constant, and C is the concentration of ammonium or nitrate.

### Statistical analysis

Significant differences between mean values were tested by one-way analysis of variance using the least significant difference test (LSD) at the 0.05 level of probability with SPSS 20.0 statistical software (SPSS Inc., Chicago, IL, USA). The logistic and Michaelis–Menten equations were fitted in Origin Pro 9.0 (Origin Lab Inc., Hampton, VA, USA), and graphs were prepared using Graph Pad Prism V. 5.0 (GraphPad Software Inc., La Jolla, CA, USA).

## Results

### Nitrogen absorption

**Figure 2:**
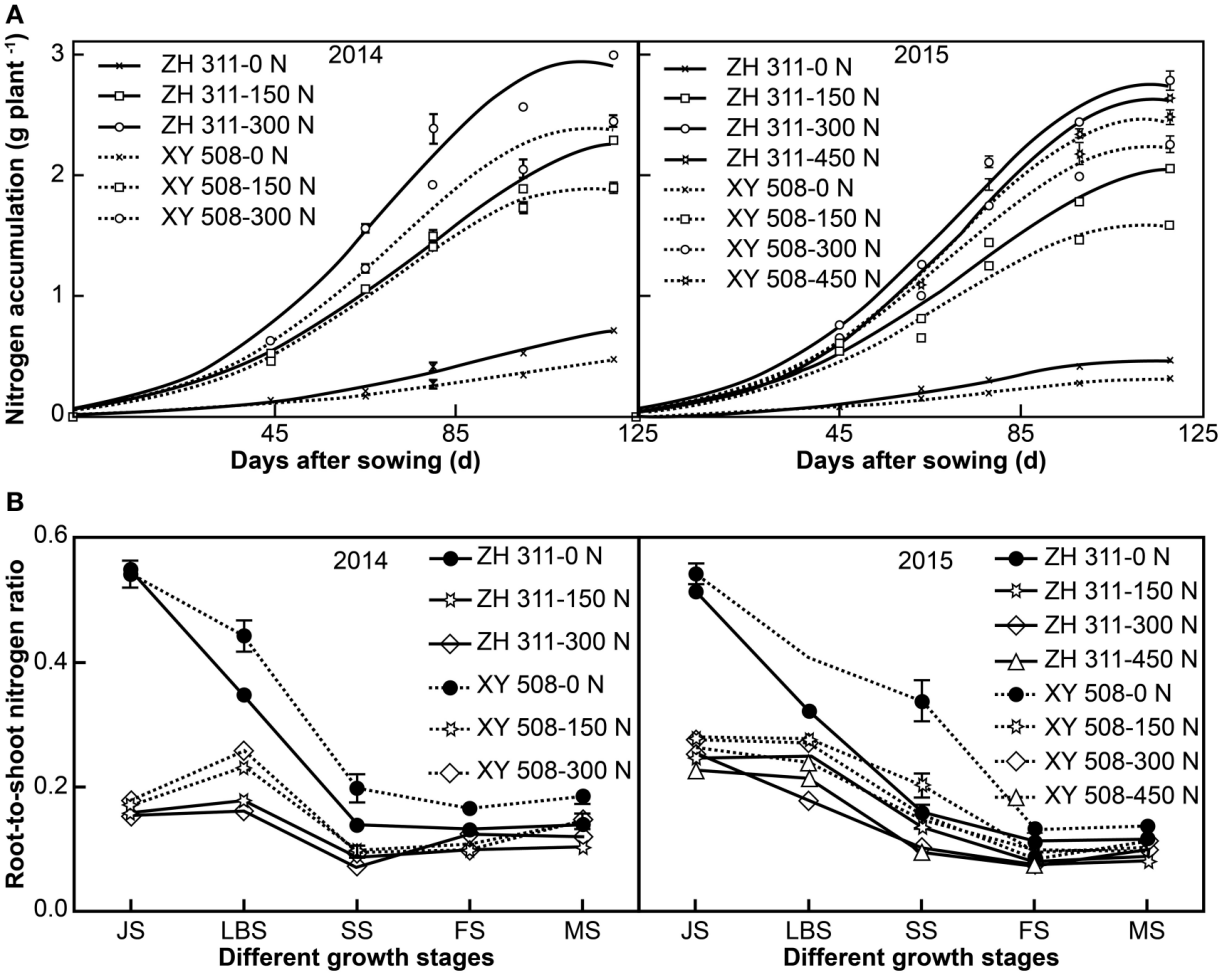
Logistic curve fitting of nitrogen absorption **(A)** and root-to-shoot nitrogen ratio in different growth stages **(B)** (Experiment I). Data are the mean ± SE of three replicate pots, with four plants per pot.

There were significant differences in N accumulation between the N treatments and maize cultivars in both years of the study (Figure 2A). The mean N accumulation in ZH 311 at JS, LBS, SS, FS, and MS was higher than that in XY 508 by 0.06, 0.14, 0.23, 0.29, and 0.39 g plant^−1^ in 2014, and by 0.05, 0.11, 0.16, 0.27, and 0.33 g plant^−1^ in 2015, respectively. N application significantly increased the N accumulation in both cultivars, although the N accumulation increment of the two cultivars was noticeably different. The N accumulation gains in ZH 311 were higher than those in XY 508 by 13.61% in 2014 and 13.09% in 2015. The differences in N accumulation between ZH 311 and XY 508 were influenced by N levels, with the largest differences being observed in treatments supplemented with 300 kg h^−1^ N. The regression equation of the N accumulation difference between ZH 311 and XY 508 (*y*) against the N level (*x*) in 2015 was *y* = −0.000008*x*^2^ + 0.003466*x* + 0.145750 (*R*^2^ = 0.9905*). The results showed that the differences in N absorption between ZH 311 and XY 508 initially increased and then decreased with increasing N levels, with the highest value being measured at an N application of 216.63 kg ha^−1^. Therefore, the N accumulation advantage of ZH 311 over XY 508 was more evident under low and moderate N levels, whereas high N levels contributed more to N accumulation in XY 508.

Maize N accumulation followed an S-shaped curve during the entire growth period and all the fitted logistic equations were significant (*R*^2^ > 0.95; Figure 2A, Table 1). Significant differences were found in the final theoretical N accumulation (*M*), maximum N uptake rate (*V*_m_), average N uptake rate (*V*_a_), time of instantaneous maximum slope (*T*_0_), and rapid N uptake period (*T*_2_–*T*_1_) under different N levels (Table 1). Compared to the treatments with 0 kg ha^−1^ N application, there were marked increases in *M, V*_m_, and *V*_a_ in treatments with N. However, *T*_0_ was advanced and *T*_2_–*T*_1_ was shortened significantly with N application. With regards to cultivar differences, there were significant differences in *M, V*_m_, *V*_m_, *T*_0_, and *T*_2_–*T*_1_ between the two cultivars. Compared with XY 508, the mean values of *M, V*_m_, and *V*_a_ for ZH 311 were higher by 24.76, 21.12, and 24.76% in 2014 and by 21.17, 16.90, and 21.17% in 2015, respectively. The *T*_0_ and *T*_2_–*T*_1_ for ZH 311 commenced later and lasted longer than those for XY 508. *T*_0_ was postponed by 6.91 and 3.92 d in 2014 and 2015, and *T*_2_–*T*_1_ was extended by 4.20 and 1.75 d in 2014 and 2015, respectively. In treatments where plants received up to 300 kg ha^−1^ of N, the N accumulation, *M, V*_m_, and *V*_a_ increased with increasing N rates in both cultivars; however, when N application exceeded 300 kg ha^−1^ in 2015, the N accumulation, *M, V*_m_, and *V*_a_ of ZH 311 plateaued and even decreased, whereas these parameters continued to increase in XY 508. In other words, the N accumulation, *M, V*_m_, and *V*_a_ of ZH 311 were fitted to N fertilizer levels with a quadratic function, whereas those of XY 508 were fitted with an approximate linear function.

**Table 1:** Logistic equation characteristics of N accumulation in different treatments in 2014 and 2015 (Experiment I).

**Cultivar**	**Nitrogen rate (kg ha^−1^)**	**Regression equation**	***R*^2^**	***M* (g plant^−1^)**	***V*_m_ (mg plant^−1^ d^−1^)**	***V*_a_ (mg plant^−1^ d^−1^)**	***T*_0_ (*d*)**	***T*_2_–*T*_1_ (*d*)**
**2014**	0 N	*y* = 0.87986/(1 + 42.18444EXP^−0.04265^)	0.9780	0.88d	9.41d	7.34d	87.02a	61.69a
ZH 311	150 N	*y* = 2.47505/(1 + 30.87484EXP ^−0.04770**x**^)	0.9938	2.48b	29.60c	20.68b	72.07b	55.30b
	300 N	*y* = 2.93293/(1 + 82.39378EXP ^−0.07065**x**^)	0.9816	2.94a	51.91a	24.48a	62.57c	37.47d
	Average			2.10A	30.31A	17.50A	74.16A	51.49A
	0 N	*y* = 0.69241/(1 + 24.40921EXP ^−0.04270**x**^)	0.9748	0.70e	5.74e	5.86e	74.82b	58.54ab
XY 508	150 N	*y* = 1.94140/(1 + 45.39570EXP ^−0.06007**x**^)	0.9984	1.94c	29.16c	16.18c	63.53c	43.87c
	300 N	*y* = 2.40577/(1 + 69.06229EXP ^−0.06681**x**^)	0.9816	2.41b	40.17b	20.05b	63.40c	39.44d
	Average			1.68B	25.02B	14.03B	67.25B	47.28B
*F*-value	Cultivar (C)			256.01^**^	30.91^**^	256.44^**^	39.79^**^	48.63^**^
	Nitrogen (N)			1886.54^**^	548.96^**^	1889.06^**^	100.41^**^	430.05^**^
	C × N			20.96^**^	12.49^**^	21.07^**^	13.83^**^	41.88^**^
**2015**	0 N	*y* = 0.48695/(1 + 42.64041EXP ^−0.05012**x**^)	0.9880	0.49g	7.68f	4.13g	74.88a	52.53a
	150 N	*y* = 2.24803/(1 + 30.52863EXP ^−0.04911**x**^)	0.9760	2.25e	27.60d	19.05e	69.61b	53.64a
ZH 311	300 N	*y* = 2.86145/(1 + 44.89225EXP ^−0.05933**x**^)	0.9868	2.86a	42.44ab	24.26a	64.15e	44.43c
	450 N	*y* = 2.73707/(1 + 64.89356EXP ^−0.06240**x**^)	0.9885	2.74b	42.70a	23.20b	66.88d	42.22d
	Average			2.08A	30.11A	17.66A	68.88A	48.20A
	0 N	*y* = 0.34676/(1 + 30.53887EXP ^−0.04991**x**^)	0.9881	0.35h	4.33g	2.94h	68.51c	52.79a
	150 N	*y* = 1.69038/(1 + 32.66548EXP ^−0.05463**x**^)	0.9535	1.69f	23.09e	14.33f	63.82e	48.22b
XY 508	300 N	*y* = 2.32867/(1 + 42.36136EXP ^−0.05888**x**^)	0.9787	2.33d	34.28c	19.75d	63.64e	44.77c
	450 N	*y* = 2.51174/(1 + 66.79792EXP ^−0.06579*x*^)	0.9847	2.51c	41.32b	21.29c	63.87e	40.06e
	Average			1.72B	25.75B	14.57B	64.96B	46.46B
*F*-value	Cultivar (C)			374.31^**^	139.16^**^	373.85^**^	302.52^**^	16.15^**^
	Nitrogen (N)			3026.76^**^	1945.16^**^	3024.10^**^	225.76^**^	154.10^**^
	C × N			31.86^**^	14.95^**^	31.85^**^	36.08^**^	9.74^**^

*Data are the means of three replicates. Values with different lowercase letters are significantly different at p < 0.05; within cultivars, values with different uppercase letters are significantly different at p < 0.05 according to the least significant difference test*.

** *p < 0.01; ns, not significant*.

The correlation and path analysis of both cultivars in the 2 years of experiments showed that *M* was significantly positively correlated with *V*_m_ and *V*_a_ and significantly negatively correlated with *T*_0_ and *T*_2_–*T*_1_. The contribution of *V*_a_ to *M* was the greatest (over 80%), followed by *V*_m_ and *T*_2_–*T*_1_. These results indicated that the primary and secondary factors responsible for differences in the N uptake and accumulation of ZH 311 and XY 508 were *V*_a_ and *T*_2_–*T*_1_, respectively.

### Root nitrogen uptake rate

The root N uptake rate decreased with the progression of maize growth stages, with the highest root N uptake rate being observed at the sowing stage–JS in both years (Table 2). The N uptake rate of ZH 311 roots was higher by 11.99% during the sowing stage–JS, 15.54% in JS–SS, 18.49% in SS–MS, and 8.28% over the entire growth period compared with that of XY 508 roots in 2014 and by 9.27, 22.82, 32.31, and 4.26% in the respective stages in 2015. These results showed that the differences in N absorption between the two cultivars mainly stem from the differences in root N uptake rate in later growth stages. The average root N uptake rate over the entire growth period in ZH 311 was higher by 16.09, 2.67, and 9.47% compared with that in XY 508 in treatments with 0, 150, and 300 kg ha^−1^ of N, respectively, in 2014, and by 23.33, 10.39, 1.04, and −4.56% in treatments with 0, 150, 300, and 450 kg ha^−1^ of N, respectively, in 2015.

**Table 2:** Root nitrogen uptake rates in different growth periods in 2014 and 2015 (Experiment I).

**Cultivar**	**Nitrogen rate (kg ha^−1^)**	**Sowing stage–jointing stage (mg g**^**−1**^ **RDW d**^**−1**^**)**		**Jointing stage–silking stage (mg g**^**−1**^ **RDW d**^**−1**^**)**		**Silking stage–maturity stage (mg g**^**−1**^ **RDW d**^**−1**^**)**		**Average (mg g**^**−1**^ **RDW d**^**−1**^**)**	
		**2014**	**2015**	**2014**	**2015**	**2014**	**2015**	**2014**	**2015**
ZH 311	0 N	1.21c	1.34c	1.15d	1.45f	0.75b	0.58d	1.01c	1.11d
	150 N	3.89b	4.03b	2.43c	2.03d	0.96a	0.81b	1.54b	1.70c
	300 N	4.61a	4.41a	4.26a	3.16b	0.70b	0.80b	1.85a	1.95b
	450 N	–	4.55a	–	3.57a	–	0.95a	–	2.09ab
	Average	3.24A	3.58A	2.61A	2.56A	0.80A	0.79A	1.47A	1.71A
XY 508	0 N	1.10c	1.38c	0.82e	0.82g	0.74b	0.48e	0.87c	0.90e
	150 N	3.69b	3.80b	2.43c	1.76e	0.66b	0.46e	1.50b	1.54c
	300 N	3.88b	4.02b	3.53b	2.62c	0.66b	0.67c	1.69ab	1.93b
	450 N	–	3.91b	–	3.12b	–	0.74b	–	2.19a
	Average	2.89B	3.28B	2.26B	2.08B	0.69B	0.59B	1.35B	1.64B
*F*-value	Cultivar (C)	6.92^*^	36.39^**^	14.59^**^	74.03^**^	16.24^**^	138.76^**^	5.69^*^	5.58^*^
	Nitrogen (N)	214.19^**^	759.51^**^	336.45^**^	322.51^**^	6.44^*^	63.85^**^	111.99^**^	270.70^**^
	C × N	2.14	8.00^**^	5.29^*^	1.88	10.51^**^	11.92^**^	0.74	5.64^**^

*RDW, root dry weight. Data are the means of three replicates. Values with different lowercase letters are significantly different at p < 0.05; within cultivars, values with different uppercase letters are significantly different at p < 0.05 according to the least significant difference test*.

** p < 0.01;

* *p < 0.05; ns, not significant*.

In both study years, significant differences were found in the root N uptake rate during all growth periods under different N treatments. At the sowing stage–JS, the root N uptake rate (*y*) increased with increasing N rates (*x*). However, at N levels exceeding 300 kg ha^−1^, the N accumulation decreased, thus following a quadratic function. Regarding the JS–SS, SS–MS, and entire growth periods, the root N uptake rate increased with increasing N rates following a linear positive correlation, whereas the increments in the root N uptake rate between the two cultivars were clearly different. The application of N fertilizer at a rate of 100 kg ha^−1^ increased the average root N uptake rate to 0.213 and 0.284 mg g^−1^ root dry weight d^−1^ in the entire growth period of ZH 311 and XY 508, respectively. The higher gain in XY 508 indicates that ZH 311 was affected less by N fertilizer and therefore was less sensitive to N fertilizer compared with XY 508, which was sensitive to N fertilization level, and particularly to N deficiency.

In addition, the average dry weight of ZH 311 roots in N treatments was slightly higher, by 9.69% in 2014 and 3.66% in 2015, than that of XY 508, whereas the dry weight of ZH 311 roots in treatments that were not supplemented with N was significantly higher, by 34.06% and 23.87% in 2014 and 2015 (data not shown), respectively, than that of XY 508 roots. These data indicate that the N-efficient cultivar ZH 311 maintained higher root dry weight, thereby enhancing its adaptability to N-deficient conditions.

### Root-to-shoot nitrogen ratio

Significant differences were also found in the root-to-shoot N ratio under different N levels in the substrate (Figure 2B). The root-to-shoot N ratio was significantly lower with N treatments than with treatments with no N application by 69.92% at JS, 47.77% at LBS, 48.37% at SS, 27.71% at FS, and 20.97% at MS in 2014, and by 51.31, 34.67, 44.21, 31.10, and 23.43%, respectively, in 2015. There were significant differences in the root-to-shoot N ratios of the two maize cultivars. The root-to-shoot N ratio of XY 508 was higher than that of ZH 311 by 3.44% at JS, 35.79% at LBS, 31.88% at SS, 4.88% at FS, and 30.90% at MS in 2014, and by 9.62, 39.56, 75.65, 20.08, and 7.87% respectively, in 2015. The differences between ZH 311 and XY 508 in the root-to-shoot N ratio at LBS and SS stages were higher than those at other stages and significantly higher in treatments with no N application than in treatments with different levels of N. Furthermore, the root-to-shoot ratio of N in ZH 311 at MS was lower by 24.08, 28.25, and 18.43% than that in XY 508 in treatments with 0, 150, and 300 kg h^−1^ of N, respectively, in 2014, and by 14.73, 19.39, 11.47, and 8.42% in treatments with 0, 150, 300, and 450 kg h^−1^ of N, respectively, in 2015. The differences between ZH 311 and XY 508 initially increased and then decreased, with the highest values being observed in treatments with 150 kg h^−1^ of N in both experimental years. These results indicate that the N-efficient cultivar was better able to coordinate the N allocation between the shoot and root, particularly at moderate N levels.

**Figure 3:**
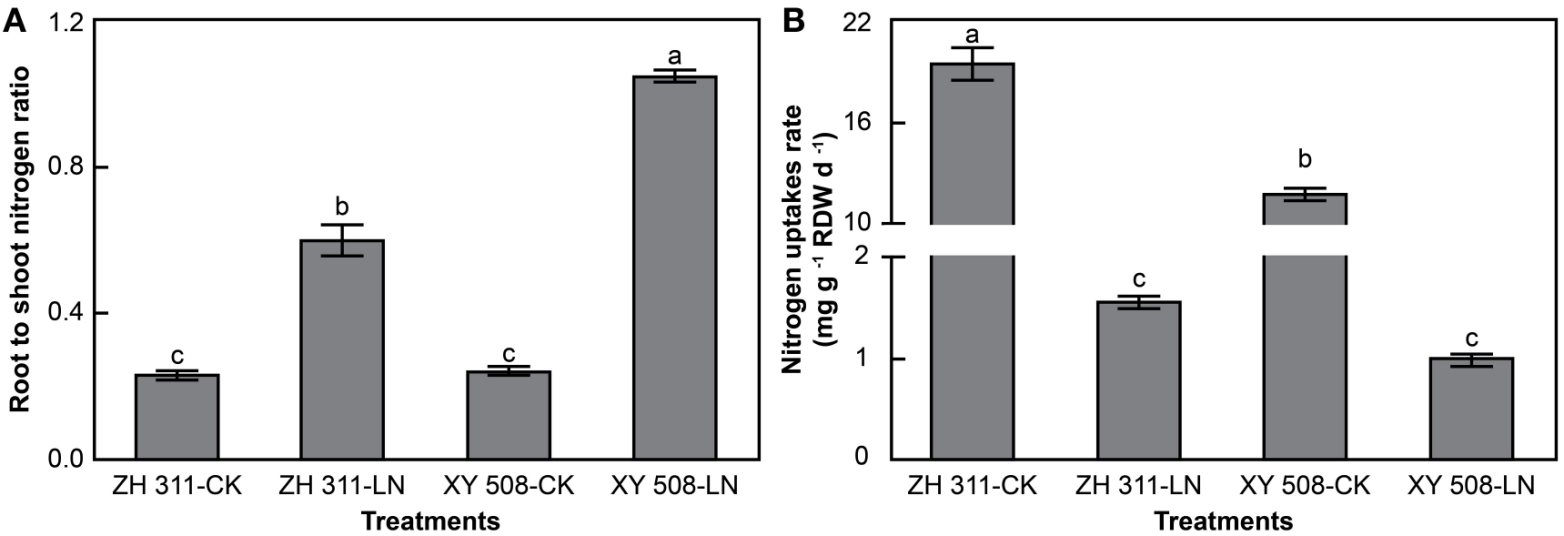
Root-to-shoot nitrogen ratio **(A)** and root nitrogen uptake rates **(B)** (Experiment II). Data are the mean ± SE of three replicate pots. Values with different lowercase letters are significantly different at *p* < 0.05.

Under LN stress, there was a significant increase in the root-to-shoot ratio of N in maize (Figure 3A), by 160.48 and 330.12% in ZH 311 and XY 508, respectively. In contrast, the root N uptake rate was significantly decreased (Figure 3B). The N uptake rate of ZH 311 roots was 65.22 and 55.23% higher than that of XY 508 roots under CK and LN treatments, respectively. This indicates that the N uptake ability of ZH 311 roots was greater than that of XY 508 roots under both normal and low N conditions, thereby indicating an enhanced coordinated distribution of N in the roots and shoots of ZH 311, particularly under low N stress.

### Kinetics of root nitrogen uptake

**Figure 4:**
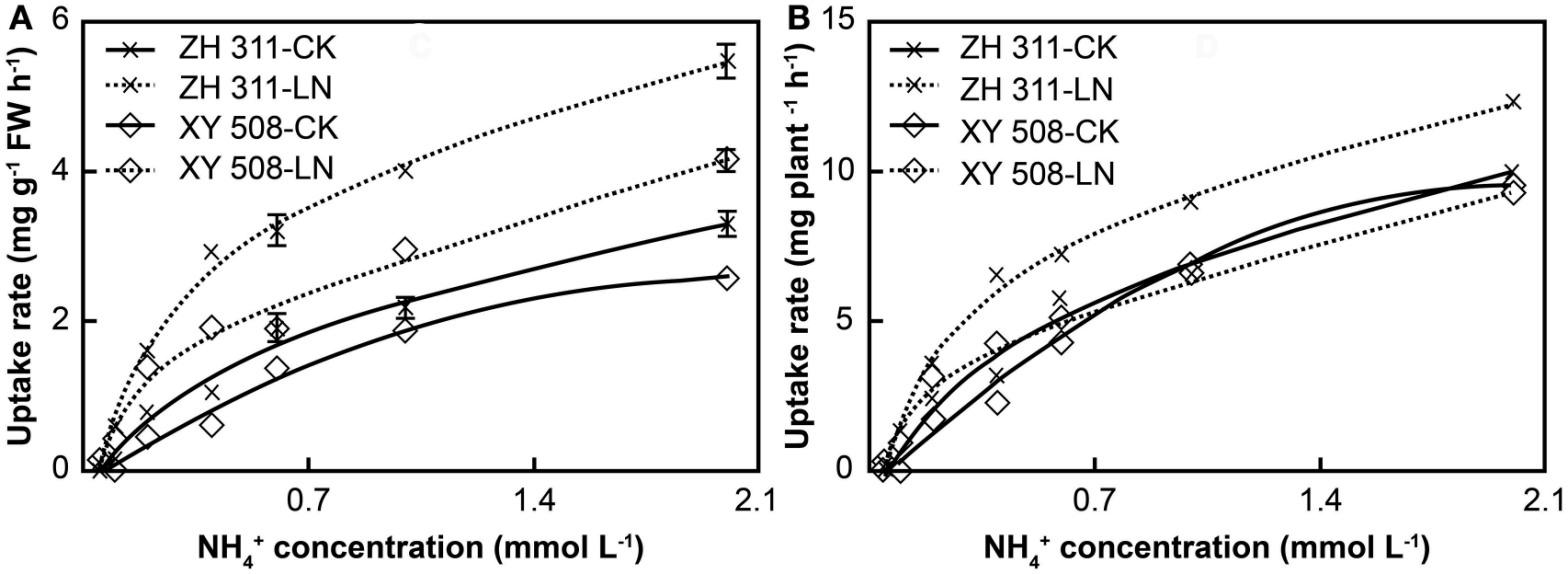
Ammonium nitrogen uptake rates of maize cultivars with contrasting nitrogen efficiency (Experiment II). FW, fresh weight. Data are the mean ± SE of three replicate pots, with five seedlings per pot. Uptake rate per FW **(A)** and uptake rate per plant **(B)**.

With increasing ammonium concentration, there were significant increases in the amount and rate of root ammonium uptake in the four treatments (Figure 4). Under LN stress, the ammonium uptake rate per fresh weight (FW) in both cultivars increased significantly, and the increment was visibly higher in ZH 311 than in XY 508. Moreover, at an ammonium concentration of 2 mmol L^−1^, the ammonium uptake rate in ZH 311 was 2.18 mg g^−1^ FW h^−1^ higher than that of the CK group, whereas that in XY 508 was higher only by 1.58 mg g^−1^ FW h^−1^. The ammonium uptake rates per plant increased in ZH 311 but decreased in XY 508.

**Figure 5:**
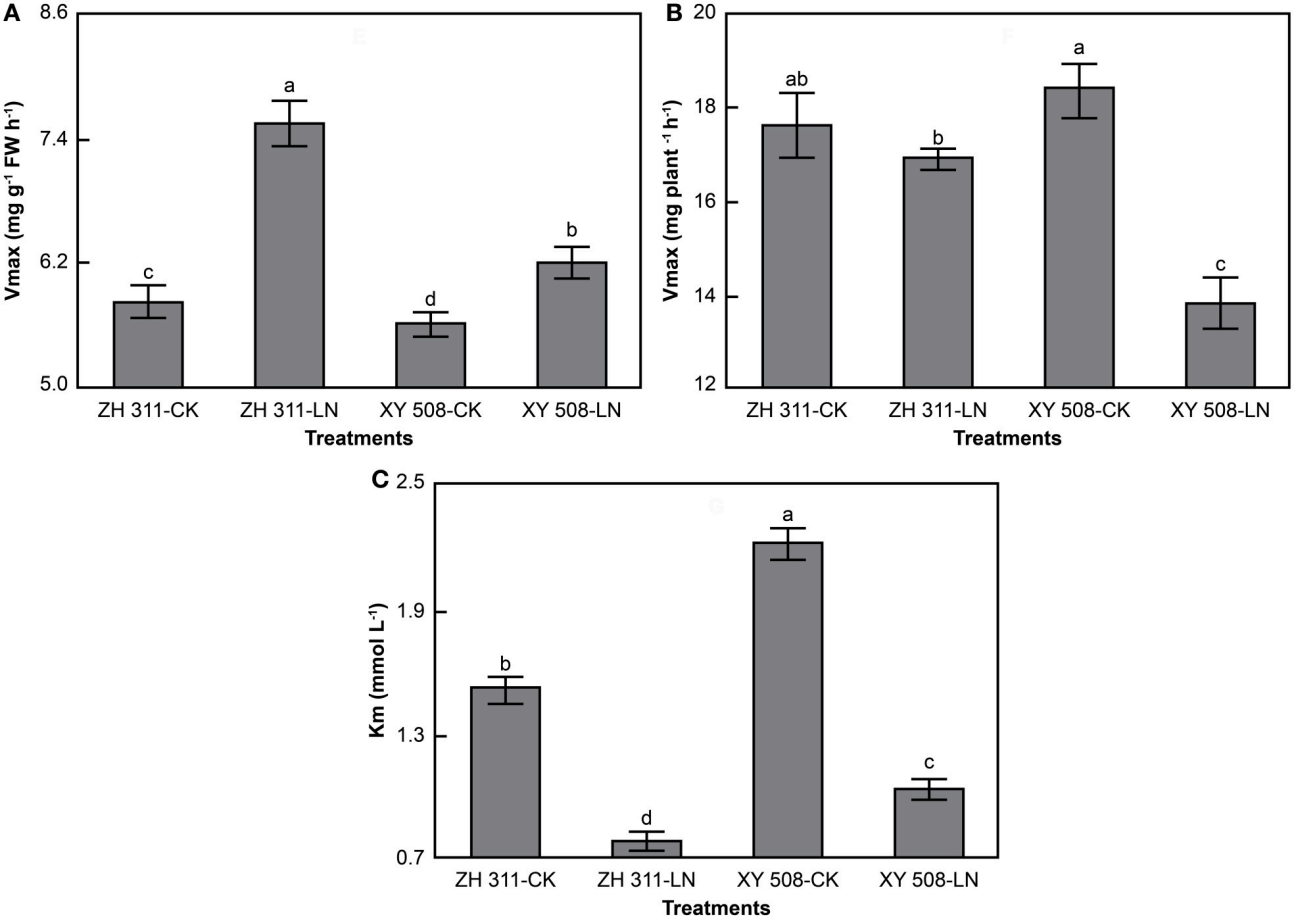
Kinetic parameters of ammonium nitrogen uptake rates in maize (Experiment II). FW, fresh weight. Data are the mean ± SE of three replicate pots. Values with different lowercase letters are significantly different at *p* < 0.05. Maximum uptake rate per FW **(A)**, maximum uptake rate per plant **(B)**, and apparent Michaelis constant **(C)**.

Data analysis showed that the root ammonium uptake rates corresponded to the Michaelis–Menten equation in all treatments (*R*^2^ > 0.96, data not shown). Significant differences were found in *V*_max_ per FW, *V*_max_ per plant, and *K*_m_ between ZH 311 and XY 508 (Figure 5). Taking into account the average values of the CK and LN groups, the *V*_max_ per FW and *V*_max_ per plant in ZH 311 were higher by 13.22% and 7.29% compared to those in XY 508, whereas *K*_m_ was lower by 29.34% compared to that in XY 508. Under LN stress, the *V*_max_ per FW was significantly increased and the *V*_max_ per plant and *K*_m_ significantly decreased in both cultivars. The increment in *V*_max_ per FW in ZH 311 was higher than that in XY 508, whereas the decrement in *V*_max_ per plant and *K*_m_ were lower than those in XY 508. These results showed that the N-efficient cultivar had a higher ammonium affinity and greater ammonium uptake potential than did the N-inefficient cultivar under both CK and LN conditions, whereas the differences in ammonium affinity and ammonium uptake potential between the N-efficient and N-inefficient cultivars were higher under LN than under CK conditions.

**Figure 6:**
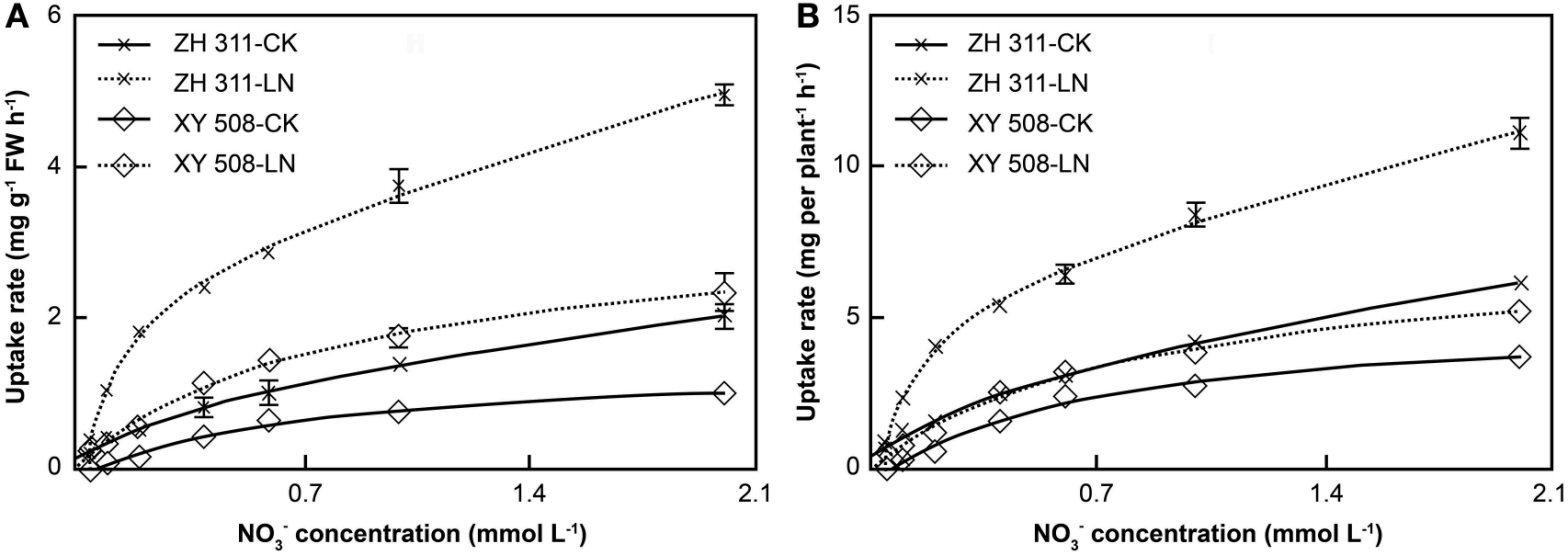
Nitrate nitrogen uptake rates of maize cultivars with contrasting nitrogen efficiency (Experiment II). FW, fresh weight. Data are the mean ± SE of three replicate pots, with five seedlings per pot. Uptake rate per FW **(A)** and uptake rate per plant **(B)**.

There were significant differences in nitrate uptake rate between the maize cultivars, being higher in the N-efficient cultivar ZH 311, particularly under LN stress (Figure 6). At a nitrate concentration of 2 mmol L^−1^, the nitrate uptake rate per FW in ZH 311 was higher than that in XY 508 by 1.03 mg g^−1^ FW h^−1^ in the CK group and by 2.61 mg g^−1^ FW h^−1^ in the LN group. The nitrate uptake rate per plant in ZH 311 was higher than that in XY 508 by 2.46 mg root^−1^ h^−1^ and 5.90 mg root^−1^ h^−1^ in the CK and LN groups, respectively. The differences in both the nitrate uptake rate per FW and nitrate uptake rate per plant between the two cultivars were higher under LN than under CK conditions, with the differences in the root nitrate uptake rate per plant being greater than those in the root nitrate uptake rate per FW. The difference between the N-efficient and N-inefficient cultivars in nitrate *V*_max_ per FW was the same as that for nitrate *V*_max_ per plant in the CK groups, whereas the difference in nitrate *V*_max_ per plant was higher than that in nitrate *V*_max_ per FW in the LN groups.

**Figure 7:**
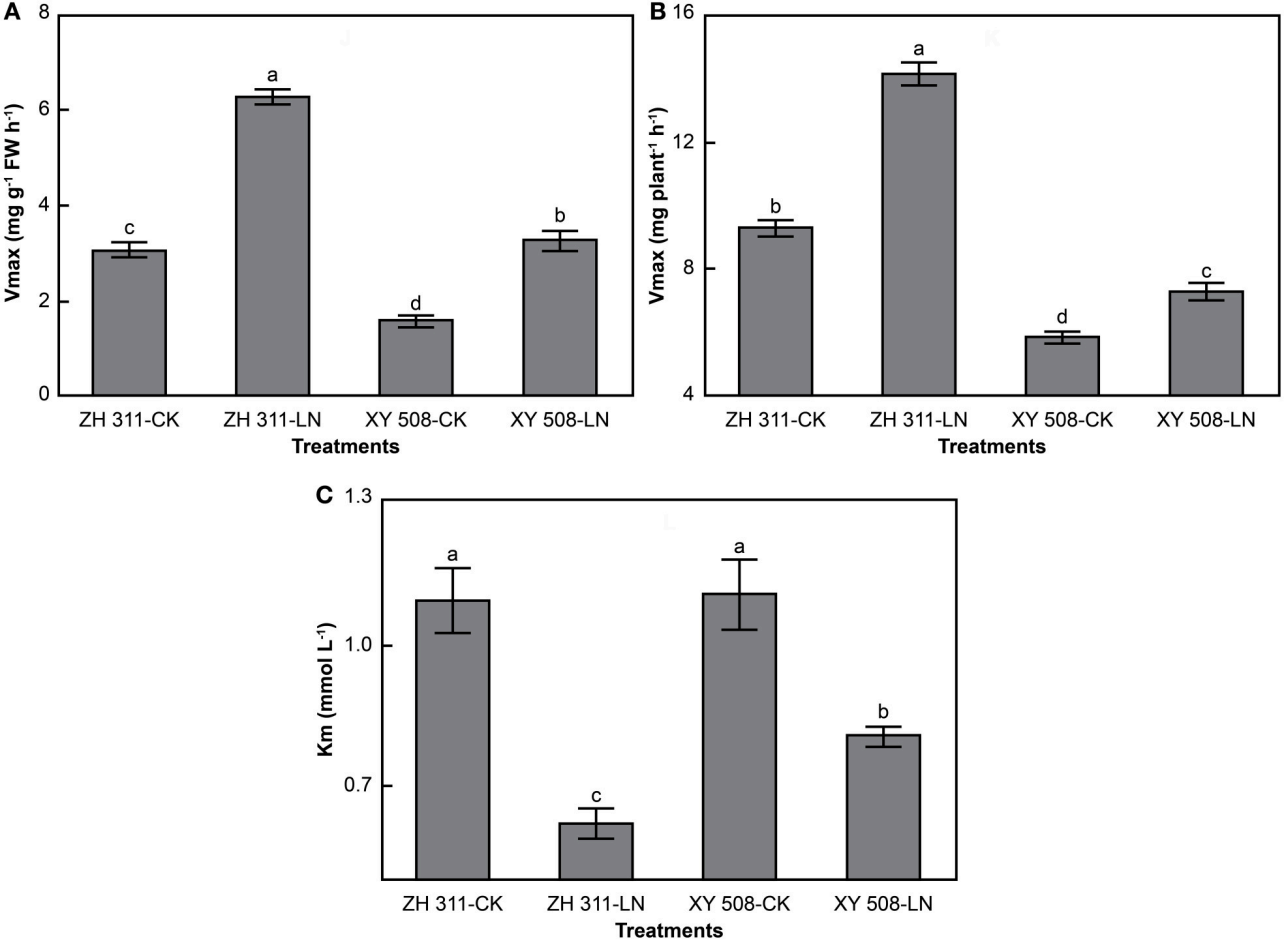
Kinetic parameters of nitrate nitrogen uptake rates of maize (Experiment II). FW, fresh weight. Data are the mean ± SE of three replicate pots. Values with different lowercase letters are significantly different at *p* < 0.05. Maximum uptake rate per FW **(A)**, maximum uptake rate per plant **(B)**, and apparent Michaelis constant **(C)**.

There were significant differences in the kinetic parameters of nitrogen uptake between maize cultivars (Figure 7). Compared with XY 508, ZH 311 showed considerably higher values for *V*_max_ per FW and *V*_max_ per plant, but lower *K*_m_, particularly under low N conditions. The *K*_m_ values for ammonium uptake were significantly higher than those for nitrate uptake—the *K*_m_ of ammonium uptake by ZH 311 and XY 508 was 34.54 and 70.58% higher, respectively, than that of nitrate uptake by the same cultivars. Furthermore, at an ionic concentration of 2 mmol L^−1^, the mean ammonium uptake rate (*V* per FW and *V* per plant) reached only 61.77 and 61.67% of the ammonium *V*_max_ per FW and *V*_max_ per plant, respectively, whereas the mean nitrate uptake rate reached 72.66 and 71.65% of the nitrate *V*_max_ per FW and *V*_max_ per plant, respectively. In terms of cultivar differences, the mean ammonium uptake rate and mean nitrate uptake rate of ZH 311 reached 65.82 and 64.63% of the *V*_max_ per FW, respectively, and 74.64 and 73.80% of the *V*_max_ per plant, respectively, whereas those of XY 508 reached 57.19 and 58.49% of the *V*_max_ per FW, and 68.85 and 67.82% of the *V*_max_ per plant, respectively. These results showed that both the nitrate affinity and nitrate uptake potential of the N-efficient cultivar are higher than those of the N-inefficient cultivar, whereas the tolerance of the N-inefficient cultivar to excessive N supply is higher than that of the N-efficient cultivar.

## Discussion

### Differences in root nitrogen uptake ability

Nitrogen is an essential component of numerous important compounds, including amino acids, proteins, nucleic acids, chlorophyll, and some plant hormones (Chun et al., 2005; Han et al., 2015). Consequently, N accumulation has a significant influence on maize growth (Liu et al., 2009), dry matter production, and yield (Mu et al., 2015). Many studies have reported the difference in N accumulation between N-efficient and N-inefficient cultivars (Worku et al., 2007; Li et al., 2010; Mu et al., 2015). Peng et al. (2010) showed that N accumulation in the whole plant of the N-efficient line 478 was significantly higher than that of the N-inefficient Wu312. Chun et al. (2005) suggested that N-efficient hybrids take up more N than N-inefficient hybrids, and that the difference in N uptake between N-efficient and N-inefficient cultivars is mainly attributed to N accumulation after silking. These results were corroborated by the present study, in which we found that the N accumulation of ZH 311 was significantly higher than that of XY 508 at both low and high N levels in two experimental systems (Figure 2, Exp. II data not shown). In addition, we also found that, compared with XY 508, the N accumulation capacity of ZH 311 was greater at low N levels than at high N concentrations (Exp. I). The correlations between N accumulation, *M, V*_m_, and *V*_a_ of ZH 311 and N levels followed a quadratic function, whereas those of XY 508 and N levels followed an approximate linear function. The greatest differences in N accumulation between ZH 311 and XY 508 was observed at low and moderate N levels, and decreased with increasing N levels. Thus, compared to the N-inefficient cultivar XY 508, the N uptake capacity of the N-efficient cultivar ZH 311 was mainly displayed under low and moderate N levels.

To acquire adequate amounts of N, plants may increase root size (root length, root volume, and root dry weight) or increase root N uptake ability (Liu et al., 2009). The absorption of N in maize was in accordance with a logistic function, whereas the curve parameters of different cultivars and N levels were clearly different. In this regard, we found that the N uptake rates *V*_m_ and *V*_a_ of ZH 311 were significantly higher than those of XY 508, whereas the N uptake times *T*_0_ and *T*_2_–*T*_1_ of ZH 311 commenced later and lasted longer than those of XY 508 (Table 1). However, the differences between ZH 311 and XY 508 in *V*_m_ and *V*_a_ were significantly higher than those pertaining to *T*_0_ and *T*_2_–*T*_1_. Therefore, differences in the N accumulation of ZH 311 and XY 508 were mainly attributable to N uptake rate rather than to uptake time. In this study, the root dry weight for ZH 311 was greater than that of XY 508, particularly under conditions of N deficiency. However, the differences between ZH 311 and XY 508 in the root N uptake ability were greater than those in the root dry weight. These results indicate that the differences in N uptake between the N-efficient cultivar ZH 311 and N-inefficient cultivar XY 508 were mainly due to the higher root N uptake ability of ZH 311, and that the greater root dry weight in ZH 311 could play a role in better N uptake under low N conditions.

In this study, the root N uptake rate decreased significantly as maize growth progressed (Table 2). The root N uptake rate of ZH 311 was significantly higher than that of XY 508 at low and high N levels in both experiments (Table 2, Figure 3B). The differences gradually increased with growth progression, and the largest difference was observed during the SS–MS period in both years (Table 2). These results are well-explained by differences in the N uptake of the N-efficient and N-inefficient cultivars that originate during the post-silking stage, which are consistent with the results reported in many previous studies (Chun et al., 2005; Li et al., 2010; Cui et al., 2013). N application significantly increased the root N uptake rate of both maize cultivars, with the increase being significantly higher in XY 508 than in ZH 311. These results showed that N application was more conducive to enhancing the root N uptake rate in XY 508, and also indicate that XY 508 is sensitive to N deficiency. Therefore, the root N uptake ability of ZH 311 was significantly higher than that of XY 508 at low and high N levels, and maintaining higher root N uptake ability is an important factor for the N-efficient cultivar ZH 311 in adaptation to N deficiency.

### Characteristics of maize root nitrogen uptake kinetics

Analysis of root uptake kinetics is the most simple and feasible approach for studying the difference in plant root uptake ability (Hu et al., 2014; Xiaochuang et al., 2015). The nutrient uptake rate of crop root systems obeys the Michaelis–Menten equation, although there are obvious differences among crops and cultivars (Ferreira et al., 2014; Zheng et al., 2016). In our study, significant differences in the uptake kinetics of ammonium and nitrate were observed between ZH 311 and XY 508 (Figures 4–7). The *K*_m_ values of ammonium and nitrate in ZH 311 were lower than those of XY 508, whereas the *V*_max_ per FW and *V*_max_ per plant of ZH 311 were higher for both N resources (Figures 5, 7). Similar results have been reported for other crops (Xiong et al., 2016; Zheng et al., 2016), with the affinity and uptake potential for ammonium and nitrate of N-efficient cultivars being shown to be significantly higher than those of N-inefficient cultivars. Furthermore, the *V*_max_ per FW and *V*_max_ per plant (except for the ammonium uptake rate in XY 508) increased significantly in both cultivars under LN stress. The increments of *V*_max_ per FW and *V*_max_ per plant in ZH 311 were markedly higher than those in XY 508, and the differences between ZH 311 and XY 508 in *V*_max_ per plant were higher than those in *V*_max_ per FW (Figures 5, 7). These results indicate that the root N uptake ability of the N-efficient cultivar ZH 311 is significantly higher than that of the N-inefficient cultivar XY 508 under both CK and LN conditions. The differences in root N uptake ability between the N-efficient cultivar ZH 311 and N-inefficient cultivar XY 508 were mainly attributable to the root N uptake rate per FW under sufficient levels of N, whereas differences under conditions of N deficiency were affected by the coordinated effects of root size and root N uptake rate per FW.

The uptake forms of N vary among crops and cultivars, since some plants preferentially take up ammonium N, whereas others tend to absorb nitrate N (Hu et al., 2014; Zheng et al., 2016). In this study, the *K*_m_ and *V*_max_ for nitrate in both cultivars were clearly lower than those for ammonium (Figures 4–7), which may be due to differences in ammonium and nitrate absorption and assimilation mechanisms. These results show that the affinity of maize roots for nitrate uptake is greater than that for ammonium uptake, that the uptake ability for nitrate is higher under low N conditions, and that nitrate is the main N uptake source in maize. The differences in the root ammonium *V*_max_ per FW and *V*_max_ per plant between ZH 311 and XY 508 were 30.30 and 18.54% at a concentration of 2 mmol L^−1^, whereas the differences in the root nitrate uptake rate were 109.01 and 93.62%, respectively (Figures 5, 7). Furthermore, the *K*_m_ values of ammonium and nitrate uptake in both cultivars decreased significantly under LN stress. Ammonium uptake by ZH 311 and XY 508 decreased by 48.58 and 53.26% and nitrate uptake by the two cultivars decreased by 42.78 and 26.89%, respectively. These results indicate that the differences in nitrate uptake between the N-efficient cultivar ZH 311 and N-inefficient cultivar XY 508 were significantly higher than those in ammonium uptake. Such disparity may be explained by the fact that nitrate is the main N source in maize.

The simplest and most effective method to reduce the application of N fertilizer in crop production is to select cultivars with higher root N uptake ability. In this study, we investigated the N uptake ability of two maize cultivars, the N-efficient ZH 311 and N-inefficient XY 508. The application of N fertilizers significantly increased N accumulation, *V*_a_, *V*_m_, and root N uptake rate, and delayed *T*_0_ and prolonged *T*_2_–*T*_1_ in both cultivars, whereas the root N uptake kinetic parameters *V*_max_ per FW and *V*_max_ per plant were significantly increased under LN stress. The *V*_a_, *V*_m_, root N uptake rate, *V*_max_ per FW, and *V*_max_ per plant of ZH 311 were significantly higher than those of XY 508, whereas the *K*_m_ of XY 508 was higher than that of ZH 311. On the basis of these observations, we can conclude that higher root N uptake ability is an important physiological mechanism in ZH 311 that enables this cultivar to efficiently acquire N, particularly under N-deficient conditions. The higher N uptake ability of the N-efficient cultivar ZH 311 compared with the N-inefficient cultivar XY 508 can mainly be attributed to the lower *K*_m_ value and higher N uptake rate of ZH 311, particularly during the later growth period. Further research on root morphology and physiology is needed to comprehensively analyze the differences between the N-efficient cultivar ZH 311 and N-inefficient cultivar XY 508 and to elucidate the efficient N uptake mechanism in N-efficient cultivars.

## Author contributions

QL and JY designed the study; QL, YW, WC, and RJ performed the experiments; QL and FK analyzed the data; YK and HS developed the new methods; and QL wrote the paper.

### Conflict of interest statement

The authors declare that the research was conducted in the absence of any commercial or financial relationships that could be construed as a potential conflict of interest.

## Abbreviations

CK: normal-nitrogen group
Exp. I and Exp. II, Experiment I and II: respectively
FS: filling stage
FW: fresh weight
JS: jointing stage
*K*_m_: Michaelis constant
LBS: large bell stage
LN: low-nitrogen group
NUE: nitrogen-use efficiency
*M*: final theoretical N accumulation
MS: maturity stage
SS: silking stage
*T*_0_: time of instantaneous maximum slope
*T*_1_: time of N uptake rate acceleration
*T*_2_: time of N uptake rate deceleration
*T*_2_–*T*_1_: rapid N uptake period
*V*: uptake rate
*V*_a_: average N uptake rate
*V*_m_: maximum N uptake rate
*V*_max_: maximum uptake rate
RDW: root dry weight.

## References

[B1] Abbasi M. K. Tahir M. M. Rahim N. (2013). Effect of N fertilizer source and timing on yield and N use efficiency of rainfed maize (*Zea mays* L.) in Kashmir–Pakistan. Geoderma 195–196, 87–93. 10.1016/j.geoderma.2012.11.013

[B2] Chen G. Chen Y. Zhao G. Cheng W. Guo S. (2015). Do high nitrogen use efficiency rice cultivars reduce nitrogen losses from paddy fields? Agric. Ecosyst. Environ. 209, 26–33. 10.1016/j.agee.2015.03.003

[B3] Chen X. Cui Z. Fan M. Vitousek P. Zhao M. Ma W.. (2014a). Producing more grain with lower environmental costs. Nature 514, 486–489. 10.1038/nature13609 25186728

[B4] Chen X. Zhang J. Chen Y. Li Q. Chen F. Yuan L. (2014b). Changes in root size and distribution in relation to nitrogen accumulation during maize breeding in China. Plant Soil 374, 121–130. 10.1007/s11104-013-1872-0

[B5] Chun L. Chen F. J. Zhang F. S. Mi G. H. (2005). Root growth, nitrogen uptake and yield formation of hybrid maize with different N efficiency. Plant Nutr. Fertilizer Sci. 11, 615–619. 10.11674/zwyf.2005.0607

[B6] Cui C. Gao J. L. Yu X. F. Wang Z. G. Sun J. Y. Hu S. P. (2013). Dry matter accumulation and nitrogen migration of high-yielding spring maize for different nitrogen efficiency in the flowering and milking stages. J. Plant Nutr. Fertilizer 19, 1337–1345. 10.11674/zwyf.2013.0607

[B7] Ferreira L. M. Rangel R. P. Tavares O. C. H. Santos L. A. Souza S. R. Fernandes M. S. (2014). Phosphorus uptake kinetics and nitrogen fractions in maize grown in nutrient solutions. Semina Ciências Agrárias 6, 2991–3001. 10.5433/1679-0359.2014v35n6p2991

[B8] Food and Agriculture Organization of the United Nations (FAO) (2012). FAOSTAT Online Database. Available online at http://faostat3.fao.org/ (Accessed May 12, 2016).

[B9] Han J. Wang L. Zheng H. Pan X. Li H. (2015). ZD958 is a low-nitrogen-efficient maize hybrid at the seedling stage among five maize and two teosinte lines. Planta 242, 935–949. 10.1007/s00425-015-2331-3 26013182

[B10] Hill C. B. Cassin A. Keeble-Gagnère G. Doblin M. S. Bacic A. Roessner U. (2016). *De novo* transcriptome assembly and analysis of differentially expressed genes of two barley genotypes reveal root-zone-specific responses to salt exposure. Sci. Rep. 6:31558. 10.1038/srep31558 27527578 PMC4985707

[B11] Hochholdinger F. Tuberosa R. (2009). Genetic and genomic dissection of maize root development and architecture. Curr. Opin. Plant Biol. 12, 172–177. 10.1016/j.pbi.2008.12.002 19157956

[B12] Hu Z. Duan S. Xu N. Mulholland M. R. (2014). Growth and nitrogen uptake kinetics in cultured *Prorocentrum donghaiense*. PLoS ONE 9:e94030. 10.1371/journal.pone.0094030 24710151 PMC3977987

[B13] Jia X. Shao L. Liu P. Zhao B. Gu L. Dong S. (2014). Effect of different nitrogen and irrigation treatments on yield and nitrate leaching of summer maize (*Zea mays* L.) under lysimeter conditions. Agric. Water Manage. 137, 92–103. 10.1016/j.agwat.2014.02.010

[B14] Koeslin-Findeklee F. Becker M. A. van der Graaff E. Roitsch T. Horst W. J. (2015). Differences between winter oilseed rape (*Brassica napus* L.) cultivars in nitrogen starvation-induced leaf senescence are governed by leaf-inherent rather than root-derived signals. J. Exp. Bot. 66, 3669–3681. 10.1093/jxb/erv170 25944925 PMC4473979

[B15] Li Q. Ma X. J. Cheng Q. B. Dou P. Yu D. H. Luo Y. H. (2016a). Effects of nitrogen application on nitrogen utilization and nitrogen balance in field of maize cultivars with different low nitrogen tolerance. J. Soil. Water Conserv. 30, 171–176. 10.13870/j.cnki.stbcxb.2016.03.000

[B16] Li Q. Ma X. J. Cheng Q. B. Dou P. Yu D. H. Luo Y. H. (2016b). Effects of nitrogen fertilizer on post-silking dry matter production and leaves function characteristics of low-nitrogen tolerance maize. Chin. J. Eco Agric. 24, 17–26. 10.13930/j.cnki.cjea.150744

[B17] Li W. J. He P. Gao Q. Jin J. Y. Hou Y. P. Yin C. X. (2010). Dry matter formation and nitrogen uptake in two maize cultivars differing in nitrogen use efficiency. Plant Nutr. Fertilizer Sci. 16, 51–57. 10.11674/zwyf.2010.0108

[B18] Liu J. X. Chen F. J. Olokhnuud C. L. Glass A. D. M. Tong Y. P. Zhang F. S. (2009). Root size and nitrogen-uptake activity in two maize (*Zea mays*) inbred lines differing in nitrogen-use efficiency. J. Plant Nutr. Soil Sci. 172, 230–236. 10.1002/jpln.200800028

[B19] Liu X. Zhang Y. Han W. Tang A. Shen J. Cui Z.. (2013). Enhanced nitrogen deposition over China. Nature 494, 459–462. 10.1038/nature11917 23426264

[B20] Moll R. H. Kamprath E. J. Jackson W. A. (1982). Analysis and interpretation of factors which contribute to efficiency of nitrogen utilization. Agron. J. 74, 562–564. 10.2134/agronj1982.00021962007400030037x

[B21] Mori A. Fukuda T. Vejchasarn P. Nestler J. Pariasca-Tanaka J. Wissuwa M. (2016). The role of root size versus root efficiency in phosphorus acquisition in rice. J. Exp. Bot. 67, 1179–1189. 10.1093/jxb/erv557 26842979

[B22] Mu X. Chen F. Wu Q. Chen Q. Wang J. Yuan L. (2015). Genetic improvement of root growth increases maize yield via enhanced post-silking nitrogen uptake. Eur. J. Agron. 63, 55–61. 10.1016/j.eja.2014.11.009

[B23] Mueller N. D. Gerber J. S. Johnston M. Ray D. K. Ramankutty N. Foley J. A. (2012). Closing yield gaps through nutrient and water management. Nature 490, 254–257. 10.1038/nature11420 22932270

[B24] National Bureau of Statistics of China (2015). Database. Available online at http://www.stats.gov.cn/ (Accessed May 12, 2016).

[B25] Peng Y. Niu J. Peng Z. Zhang F. Li C. (2010). Shoot growth potential drives N uptake in maize plants and correlates with root growth in the soil. Field Crop Res. 115, 85–93. 10.1016/j.fcr.2009.10.006

[B26] Presterl T. Groh S. Landbeck M. Seitz G. Schmidt W. Geiger H. H. (2002). Nitrogen uptake and utilization efficiency of European maize hybrids developed under conditions of low and high nitrogen input. Plant Breed. 121, 480–486. 10.1046/j.1439-0523.2002.00770.x

[B27] Schnable P. S. Ware D. Fulton R. S. Stein J. C. Wei F. Pasternak S.. (2009). The B73 maize genome: complexity, diversity, and dynamics. Science 326, 1112–1115. 10.1126/science.1178534 19965430

[B28] Sepaskhah A. R. Fahandezh-Saadi S. Zand-Parsa S. (2011). Logistic model application for prediction of maize yield under water and nitrogen management. Agric. Water Manage. 99, 51–57. 10.1016/j.agwat.2011.07.019

[B29] Sheehy J. E. Mitchell P. L. Allen L. H. Ferrer A. B. (2006). Mathematical consequences of using various empirical expressions of crop yield as a function of temperature. Field Crops Res. 98, 216–221. 10.1016/j.fcr.2006.02.008

[B30] Singh P. Agrawal M. Agrawal S. B. Singh S. Singh A. (2015). Genotypic differences in utilization of nutrients in wheat under ambient ozone concentrations: growth, biomass and yield. Agric. Ecosyst. Environ. 199, 26–33. 10.1016/j.agee.2014.07.021

[B31] Tiemens-Hulscher M. Lammerts Van Bueren E. T. Struik P. C. (2014). Identifying nitrogen-efficient potato cultivars for organic farming. Euphytica 199, 137–154. 10.1007/s10681-014-1143-z

[B32] Tilman D. Balzer C. Hill J. Befort B. L. (2011). Global food demand and the sustainable intensification of agriculture. Proc. Natl. Acad. Sci. U.S.A. 108, 20260–20264. 10.1073/pnas.1116437108 22106295 PMC3250154

[B33] Tsukagoshi H. (2016). Control of root growth and development by reactive oxygen species. Curr. Opin. Plant Biol. 29, 57–63. 10.1016/j.pbi.2015.10.012 26724502

[B34] Vitousek P. M. Naylor R. Crews T. David M. B. Drinkwater L. E. Holland E.. (2009). Nutrient imbalances in agricultural development. Science 324, 1519–1520. 10.1126/science.1170261 19541981

[B35] Wang Y. Mi G. Chen F. Zhang J. Zhang F. (2004). Response of root morphology to nitrate supply and its contribution to nitrogen accumulation in maize. J. Plant Nutr. 27, 2189–2202. 10.1081/PLN-200034683

[B36] Worku M. Bänziger M. Erley G. S. A. M. Friesen D. Diallo A. O. Horst W. J. (2007). Nitrogen uptake and utilization in contrasting nitrogen efficient tropical maize hybrids. Crop Sci. 47, 519–528. 10.2135/cropsci2005.05.0070

[B37] Xiaochuang C. Lianghuan W. Ling Y. Xiaoyan L. Yuanhong Z. Qianyu J. (2015). Uptake and uptake kinetics of nitrate, ammonium and glycine by pakchoi seedlings (*Brassica campestris* L. ssp. chinensis L. Makino). Sci. Hortic. 186, 247–253. 10.1016/j.scienta.2015.02.010

[B38] Xiong S. P. Wu K. Y. Wang X. C. Wu Y. X. Du P. Ma X. M. (2016). Analysis of nitrogen metabolism in roots and uptake characteristic of wheat cultivars with different nitrogen efficiency at seedling stage. J. Trit. Crops 49, 2267–2279. 10.3864/j.issn.0578-1752.2016.12.003

[B39] Xu Z. X. Duan S. S. Xu N. Mulholland M. R. (2014). Growth and nitrogen uptake kinetics in cultured *Prorocentrum donghaiense*. PLoS ONE 9:e94030. 10.1371/journal.pone.0094030 24710151 PMC3977987

[B40] Yan D. C. Zhu Y. Wang S. H. Cao W. X. (2006). A quantitative knowledge-based model for designing suitable growth dynamics in rice. Plant Prod. Sci. 9, 93–105. 10.1626/pps.9.93

[B41] Yin G. H. Gu J. Zhang F. S. Hao L. Cong P. F. Liu Z. X. (2014). Maize yield response to water supply and fertilizer input in a semi-arid environment of Northeast China. PLoS ONE 9:e86099. 10.1371/journal.pone.0086099 24465896 PMC3896526

[B42] Zhao B. Dong S. T. Zhang J. W. Liu P. (2013). Effects of controlled-release fertiliser on nitrogen use efficiency in summer maize. PLoS ONE 8:e70569. 10.1371/journal.pone.0070569 23936449 PMC3732217

[B43] Zheng S. L. Cheng H. Li P. H. Yuan J. C. (2016). Root vigor and kinetic characteristics and nitrogen use efficiencies of different potato (*Solanum tuberosum* L.) cultivars. J. Agric. Sci. Technol. 18, 399–410.

